# Determinants of Health-Seeking Behavior in Major Depressive Disorder: An Observational Study

**DOI:** 10.7759/cureus.41901

**Published:** 2023-07-14

**Authors:** Vedant Desai, Priti Solanky, Viren Solanki, Hemal Makwana, Harshit Raj, Hetanshi M Patel

**Affiliations:** 1 Medical School, Gujarat Medical Education and Research Society (GMERS) Medical College and Hospital Valsad, Valsad, IND; 2 Preventive Medicine, Gujarat Medical Education and Research Society (GMERS) Medical College and Hospital Valsad, Valsad, IND; 3 Psychiatry, Gujarat Medical Education and Research Society (GMERS) Medical College and Hospital Valsad, Valsad, IND

**Keywords:** demographic variation, depression stigma, community awareness, mdd (major depressive disorder), delayed help-seeking behavior

## Abstract

Background: Major depressive disorder (MDD) is one of the most common illnesses in the world and a major cause of years lived with disability. It is necessary to diagnose and treat depression promptly.

Objectives: To identify and compare factors affecting health-seeking behavior in patients suffering from MDD.

Methods: An observational cross-sectional study was conducted. The study population was divided into two groups: early and late health seekers (cut off: three months). Patient Health Questionnaire - 9 (PHQ-9) as well as Perceived and Personal Depression Stigma Scores were calculated. Data were analyzed and the chi-square test and z-test were used to calculate statistical significance.

Results: There were 102 participants. The majority were female (62.75%) and the maximum number of participants were from the age group of 26-45 years (65.69%). There were more early help seekers (61.76%) than late help seekers (38.24%). The majority of early help seekers were married individuals. Distance played a vital role in help-seeking behavior. A significant association was also found between participants’ personal stigma and late treatment seeking. The most common reason for delaying medical attention was that patients thought that they could cure themselves, followed by a lack of awareness.

Conclusion: Delay and hesitance observed concerning health-seeking behavior are assumed to be associated with factors such as gender, income, family or marital status, stigma, lack of awareness, beliefs and practices, and deficient health facilities causing delays in the diagnosis and management of MDD. The research supported that involving primary health care centers, spreading awareness about the disease, and increasing psychiatric facilities, along with a special emphasis on factors as mentioned like gender, marital status, stigma, and feasibility of reaching facility as distance plays a major role in causing delay, and can help decrease the duration of symptom from the onset, initiating appropriate treatment, and improving prognosis.

## Introduction

Depression, also known as major depressive disorder (MDD), is a mood disorder characterized by low mood, lack of energy, persistent sadness, sleep disturbance, fatigue, and absence of interest in previously rewarding or enjoyable activities. It affects all ages, socio-economic statuses, or gender (more prevalent in women than men) globally [1,2].

Various causes of depression can be genetic, environmental, personality, or biochemical [3]. It has a lifetime prevalence of 5.25% in India [2]. The overall point prevalence of MDD was 4.7% and pooled annual incidence was 3% [4]. Globally, depression is one of the most common illnesses with 3.8% of the population affected. Among them 5.0% are adults and 5.7% are older than 60 years [1]. Depression, in terms of YLDs (years lost to disability), is 4.33%, being the leading cause of disability worldwide, as reported by WHO during a Global Health Estimates report in 2017 [5]. Regarding the current experience of depressive disorders, the rates for females (3.0%) were slightly higher compared to that for males (2.3%). In Gujarat, the prevalence was found to be 1.3% for depressive disorder with a treatment gap of 66.7% [2].

Treatments exist for MDD. However, treatment and support services for depression are often absent or underdeveloped in low- and middle-income countries. It is found that 75% of individuals affected with mental disorders in these countries do not receive any treatment [1].

The gap in treatment or no treatment for MDD affects the efficiency of people in life, creating challenges at work, at college, and even in their families, which eventually leads to suicidal tendencies [1].

Despite decades of evidence-based interventions, depression remains a challenge for public health, due to the large treatment gap and lag. This to some extent results from low professional help-seeking by people having depressive symptoms [6].

The rates of treatment-seeking behavior are quite low among the rural population for depressive disorders. One study found that a large proportion of the participants (approximately 86%) who screened positive for depression did not seek out treatment, contributing to the treatment gap. Urban and rural areas both had a similar prevalence of depression but the larger treatment gap is from the rural agricultural community [7-9].

Various factors are identified to affect help-seeking behavior and one model which attempted to review them systematically is Andersen’s behavioral model of healthcare utilization [10], which implies three groups of determinants of help-seeking: (1) predisposing factors, i.e. factors referring to individuals that existed before their illness, like age, gender, and other sociodemographic variables; (2) enabling factors; factors that affect access to mental state care, like the numbers and geographical distribution of healthcare institutions, costs involved in health care, and financial situation of help seekers; (3) need factors, referring to “evaluated need for care” and “perceived need for care,” which are the professional opinion regarding an individual’s requirement for healthcare contact and a person’s individual opinion about the requirement for help-seeking, respectively [10].

As per a study by Valipay et al., the factors that led to late help-seeking in MDD were socio-demographic factors such as higher family income and joint family type, as well as rural locality and patient-related factors such as higher levels of perceived stigma. Family type, stigma, and living in a country with a lack of awareness were found to be significant factors related to delayed help-seeking [5].

This study was conducted in Valsad, a district on the border of the South Gujarat region with a predominantly rural and tribal population (62.74%), keeping in mind the beliefs and cultural influence of these communities affecting the necessity for health-seeking, especially with regards to mental health [11].

The objective of this study is to identify factors affecting help-seeking behavior among patients of MDD and to compare socio-demographic and behavioral factors among early and late treatment seekers.

## Materials and methods

The study was conducted at the Government Medical Education and Research Society (GMERS) Medical College Valsad in the state of Gujarat, India. This was a cross-sectional, questionnaire-based, observational study. The duration of data collection was from September to October 2022. A simple random sampling was used. The sample size was calculated using standard methods using the formula:

$n= (Z^2P(1-P))/d^2$

Where n = sample size of the simple random sample; Z = standard normal deviate; P = prevalence; d = allowable error 

The net prevalence of MDD in India was reported 5.8-7.3% [12]. The Valsad district population percentage of the rural and urban was 37.26% and 62.74% respectively [13]. A total of 80 patients were calculated as per the above calculation. 

The inclusion criterion of this study was an adult patient diagnosed with MDD as per DSM-5 (Diagnostic and Statistical Manual of Mental Disorders, 5th Edition) and ICD-11 (International Classification of Diseases, 11th Edition) criteria. The patients who refused to participate were excluded from this study. The tool used in this study was a semi-structured questionnaire to collect basic socio-demographic information, details of disease onset, health care, facilitators, and barriers to health care affecting health care access. To assess the depressive state and severity of depression of participants, the PHQ-9 questionnaire was used. It is validated for use in primary care (Table 1). The total score is interpreted as minimal depression (1-4), mild depression (5-9), moderate depression (10-14), moderately severe depression (15-19), and severe depression (20-27) [14,15]. 

**Table 1:** Patient Health Questionnaire. For scoring, add together the column score to get the total score.

	Over the last 2 weeks, how often have you been bothered by any of the following problems?	Not at all	Several days	More than half of the days	Nearly everyday
1	Little interest or pleasure in doing things.	0	1	2	3
2	Feeling down, depressed, or hopeless.	0	1	2	3
3	Trouble falling or staying asleep, or sleeping too much.	0	1	2	3
4	Feeling tired, or having little energy.	0	1	2	3
5	Poor appetite or overeating.	0	1	2	3
6	Feeling bad about yourself or that you are a failure or have let yourself or your family down.	0	1	2	3
7	Trouble concentrating on things, such as reading the newspaper or watching television.	0	1	2	3
8	Moving or speaking so slowly that other people could have noticed. Or the opposite - being so fidgety or restless that you have been moving around a lot more than usual.	0	1	2	3
9	Thoughts that you would be better off dead, or of hurting yourself.	0	1	2	3

DSS (Depression Stigma Scale) was used to measure the stigma associated with depression. It has two subscales that measure two different types of stigmas: personal stigma (Table 2) and perceived stigma (Table 3. DSS is a five-point Likert scale with a minimum score of 0 and a maximum score of 36. Responses to each item are measured. Higher scores indicate higher levels of stigma [16,17]. These scales were translated into vernacular language. They were pilot-tested and validated with senior experienced experts.

**Table 2:** Personal stigma subscale.

	Personal stigma items	Strongly disagree	Disagree	Neither disagree nor agree	Agree	Strongly Agree
1	People with depression could snap out of it if they wanted.	0	1	2	3	4
2	Depression is a sign of personal weakness.	0	1	2	3	4
3	Depression is not a real medical illness.	0	1	2	3	4
4	People with depression are dangerous.	0	1	2	3	4
5	It is best to avoid people with depression so you don't become depressed yourself.	0	1	2	3	4
6	People with depression are unpredictable.	0	1	2	3	4
7	If I had depression I would not tell anyone.	0	1	2	3	4
8	I would not employ someone if I knew they had been depressed.	0	1	2	3	4
9	I would not vote for a politician if I knew they had been depressed.	0	1	2	3	4

**Table 3:** Perceived stigma subscale.

	Perceived stigma items	Strongly disagree	Disagree	Neither disagree nor agree	Agree	Strongly Agree
1	Most people believe that people with depression could snap out of it if they wanted.	0	1	2	3	4
2	Most people believe that depression is a sign of personal weakness.	0	1	2	3	4
3	Most people believe that depression is not a medical illness.	0	1	2	3	4
4	Most people believe that people with depression are dangerous.	0	1	2	3	4
5	Most people believe that it is best to avoid people with depression so that you don't become depressed yourself.	0	1	2	3	4
6	Most people believe that people with depression are unpredictable.	0	1	2	3	4
7	If they had depression, most people would not tell anyone.	0	1	2	3	4
8	Most people would not employ someone they knew had been depressed.	0	1	2	3	4
9	Most people would not vote for a politician they knew had been depressed.	0	1	2	3	4

Proper training and sensitization of the investigator by the guide before starting the process of data collection was done. Patients were interviewed after being assessed by an on-duty psychiatrist for the competence of study participants for data collection. Approval from IHEC (Institutional Human Ethics Committee) was obtained before initiating the study. Written informed consent from every study participant was taken before collecting data. Due care was taken to maintain the confidentiality of information provided by the study participants.

Data were entered and analyzed using a Microsoft Excel sheet. Quantitative data are presented in frequency tables/graphs and proportions. The chi-square test and z-test were used to determine the statistically significant association of the variables.

## Results

This study included a sample size of 102 participants diagnosed with MDD out of which 63 (61.76%) were early help seekers and the remaining 39 (38.24%) participants were late help seekers.

Out of the total study participants, 38 (37.25%) were males and 64 (62.75%) were females. Participants were grouped according to their marital status (currently married, single, widowed or divorced). Out of the total participants, the majority of the participants were married (81; 79.41%) while the current single proportion was eight (20.59%). In this study a maximum number of participants belonged to the Hindu religion, accounting for 89.21% while the remaining 10.79% of participants belonged to the Muslim religion. There were no participants from other religions. Around 47.06% of the study participants were living in an urban area while the remaining 52.94% were from the rural area. Out of the total participants, 38 (37.25%) participants belonged to nuclear families and 64 (62.75%) participants belonged to the joint family (Table 4).

**Table 4:** Distribution of participants according to different parameters

Parameters		Total (%)
Treatment seeking behavior	Early treatment seekers	63 (61.76)
	Late treatment seekers	39 (38.24)
Gender	Male	38 (37.25)
	Female	64 (62.75)
Marital status	Married	81 (79.41)
	Currently single	21 (20.59)
Religion	Hindu	91 (89.21)
	Muslim	11 (10.79)
Locality	Urban	48 (47.06)
	Rural	54 (52.94)
Family type	Nuclear	38 (37.25)
	Joint	64 (62.75)

Out of the total 102 participants, the majority of early (61.19%) and late (38.81%) help seekers belonged to the age group 26-45 years. There were 38 male participants in the study out of which 47.37% were early help seekers while the proportion of early help-seeking was higher among female participants (70.31%). There was a significant association found between help-seeking behavior and gender (P = 0.02114) (Table 5).

A majority (69.14%) of the married participants were early help seekers while the proportion of late help-seeking was higher (66.67%) among the currently single participants (single/ divorced/ widowed). This association between marital status and help-seeking behavior was found to be statistically significant (P = 0.00263). Among the early treatment seekers, the majority belonged to the Hindu religion (60.44%), and a similar trend was reflected in late treatment seeking too, with 36.56% belonging to the Hindu religion (Table 5).

Among rural participants, 62.96% were early help seekers, and among urban participants, 60.42% were early help seekers. Among participants living in nuclear families, the majority (73.68%) were early help seekers while among participants living in joint families, this proportion of early treatment seekers was 54.69% (Table 5).

The majority of the participants in this lived within a 40 km radius of Civil Hospital Valsad and among them, 69.23% were early help seekers. Among participants living within the range of 41-80 km, 61.90% of them were early help seekers. Participants living more than 80 km away or beyond 120 km, showed late help-seeking behavior. Around 66.67% of participants who stayed further than 120 km were late help seekers. There is a statistically significant association between help-seeking behavior and distance from Civil Hospital Valsad (P = 0.04049). Among the early help seekers, the proportion of those with a secondary-level education was the highest 75% while among late help seekers, it was 25%. This difference found between help-seeking behavior and the educational qualification of participants was statistically not significant (Table 5).

Participants were divided into socio-economic classes according to a modified version of Prasad’s classification into classes I-V. As the data were collected in September and October, AICPI (All India Consumer Price Index) was taken from January 2022 as 125.1, as per the Labor Bureau, and from this AICPI, SEC (socio-economic class) was calculated. Considering the class-wise proportion of treatment-seeking, the highest early treatment seekers belonged to class IV (67.65%) and the lowest late treatment seekers also belonged to class IV. This observation was contrary to a common belief that the higher the SEC, the better will be the treatment-seeking behavior (Table 5).

**Table 5:** Distribution of participants according to their help-seeking behavior and socio-demographic profile Chi-square test

Parameter		Early treatment seekers (%)	Late treatment seekers (%)	Total (%)	x^2^	P value
Age	18-25	03 (50.00)	03 (50.00)	06 (05.88)	0.545259	0.90884
	26-45	41 (61.19)	26 (38.81)	67 (65.69)		
	46-59	11 (64.71)	06 (35.29)	17 (16.67)		
	> 60	08 (66.67)	04 (33.33)	12 (11.76)		
Gender	Male	18 (47.37)	20 (52.63)	38 (37.25)	5.314958	0.02114
	Female	45 (70.31)	19 (29.69)	64 (62.75)		
Marital status	Married	56 (69.14)	25 (30.86)	81 (79.41)	9.051599	0.00263
	Currently single	07 (33.33)	14 (66.67)	21 (20.59)		
Religion	Hindu	55 (60.44)	36 (39.56)	91 (89.22)	0.627438	0.42830
	Muslim	08 (72.73)	03 (27.27)	11 (10.78)		
Locality	Urban	29 (60.42)	19 (39.58)	48 (47.06)	0.069767	0.79168
	Rural	34 (62.96)	20 (37.04)	54 (52.94)		
Family type	Nuclear	28 (73.68)	10 (26.32)	38 (37.25)	3.643472	0.05629
	Joint	35 (54.69)	29 (45.31)	64 (62.75)		
Distance from Civil Hospital Valsad	0-40	45 (69.23)	20 (30.77)	65 (63.73)	8.284104	0.04049
	41-80	13 (61.90)	08 (38.10)	21 (20.59)		
	81-120	00 (00.00)	1 (100.00)	01 (00.98)		
	> 120	05 (33.33)	10 (66.67)	15 (14.71)		
Education	Illiterate/ primary	08 (50.00)	08 (50.00)	16 (15.69)	5.377955	0.14612
	Secondary	30 (75.00)	10 (25.00)	40 (39.22)		
	Higher secondary	12 (50.00)	12 (50.00)	24 (23.53)		
	Graduate/ above graduate	13 (59.09)	09 (40.91)	22 (21.57)		
Socio-economic class	I	12 (66.67)	06 (33.33)	18 (17.65)	2.0972	0.71789
	II	10 (50.00)	10 (50.00)	20 (19.61)		
	III	09 (64.29)	05 (35.71)	14 (13.73)		
	IV	23 (67.65)	11 (32.35)	34 (33.33)		
	V	09 (56.25)	07 (43.75)	16 (15.69)		

Participants were divided into five groups according to their severity of depression for which the PHQ-9 scale was used to measure it. The groups were divided according to the scores, which were 0-4 for no depression, 5-9 for mild depressive symptoms, 10-14 for moderate depressive symptoms, 15-19 for moderately severe symptoms of depression, and 20-27 for severe depressive symptoms. There was no statistical significance between help-seeking behavior and the severity of depression (Table 6).

**Table 6:** Comparison of PHQ-9 scores among early and late treatment seekers Chi-square test

Parameter			Early treatment seekers (%)	Late treatment seekers (%)	Total (%)	x^2^	P value
PHQ-9	Score	Severity					
	0-4	no depression	03 (50.00)	03 (50.00)	06 (05.88)	5.516661	0.23827
	5-9	mild	13 (50.00)	13 (50.00)	26 (25.49)		
	10-14	moderate	16 (61.54)	10 (38.46)	26 (25.49)		
	15-19	moderately severe	20 (80.00)	05 (20.00)	25 (24.51)		
	20-27	severe	11 (57.89)	08 (42.11)	19 (18.63)		

No statistically significant association was found between early and late help-seeking and mean perceived DSS score. However, there was a statistically significant association between early and late help-seeking and mean personal DSS scores, with the scores being higher in late help-seekers (P = 0.00097) (Table 7).

**Table 7:** Comparison of mean depression stigma score among early and late treatment seekers Z-test

DSS (Depression Stigma Scale)	Group	Mean (SD)	P value
Personal stigma sub-scale	Early treatment seekers	17.37097 (6.705109)	0.00097
	Late treatment seekers	21.475 (6.080602)	
Perceived stigma sub-scale	Early treatment seekers	21.16129 (6.709899)	0.09323
	Late treatment seekers	22.525 (5.830897)	

All participants were asked about their reasons for delaying help-seeking and we found a list of reasons such as they thought that they could get cured on their own, lack of knowledge/ awareness about their condition, social stigmas like ‘what will people think?’ and financial reasons were a major issue for most of the participants. Besides this lack or limited healthcare facility available in their locality, treatment of other diseases (as physical conditions take priority), lack of need, faith in faith healers (as this is culturally prevalent in some rural areas of Valsad district), unavailability of doctor in their locality, lack of medicine in their locality, feelings of shame when coming to psychiatrist office, and faith in ayurveda (an alternative medicine tradition originating in the Indian subcontinent) were some of the other reasons for the delay in help-seeking. For some participants, there was no reason at all to seek help late and yet they did (Table 8).

**Table 8:** Reasons for delay in help-seeking (as responded by the study participants). *multiple responses

Reasons for delay in help-seeking.	Frequency*
Thought you could cure yourself.	52
Lack of knowledge/awareness about the condition.	48
Treatment of other diseases.	8
Social stigmata: what will people think?	14
Financial reasons.	12
Unavailability of doctor in locality.	4
Lack of medicine in locality.	2
Lack or limited or low quality of healthcare available in locality.	10
Lack of need.	8
Felt shame in coming to the psychiatrist's office.	2
Faith in ayurveda.	2
Faith in faith healer.	6
None.	10

## Discussion

This study aimed to examine factors affecting help-seeking behavior among people with depression, by investigating the relationship between sociodemographic factors and patient-related factors with early or late help-seeking. Out of the total study participants majority of them i.e., 61.76%, were early treatment seekers as opposed to 38.24% who were late treatment seekers.

Age

There was no statistically significant association found between the different age groups and help-seeking behavior of the participants. This finding is consistent with the study done by Valipay et al [5].

Gender

In this study, 47.37% of the male participants were early help seekers, and 52.63% remaining male participants were late help seekers. The proportion of early help-seeking was higher among female participants (70.31%). There was a statistically significant association between gender and help-seeking. This is not consistent with the study by Valipay et al [5]. A study on depression care in the United States discovered a favorable relationship between the female gender and help-seeking. This is in line with earlier research, which discovered that males’ request for assistance was viewed as a challenge to hegemonic masculinity [18]. However, no association between sex and help-seeking was discovered by Boerema et al [19].

Marital status

Our study found a significant association between marital status and help-seeking behavior. This finding is not consistent with the other studies done by Sussman et al [20] and Boerema et al [19]. Our study suggests that unmarried individuals were more likely to be late help seekers than married participants as opposed to the study done by Coryell et al., who found a negative association between marital status but found that single individuals were more likely to seek help than married individuals [21].

Education

There was no statistically significant association found between educational level and help-seeking behavior. This observation is consistent with the study by Coryell et al. [21], whereas the study by González et al. shows a significant association between educational status and help-seeking behavior [18]. A peculiar finding in our study is that the majority of participants who were educated up to the secondary level were early help seekers. A possible explanation for this could be that individuals with higher educational status might seek help in private clinics and civil hospitals; Valsad is the only government facility in this district that provide psychiatric treatments; therefore, more individuals with fewer educational qualifications seek help here [22].

Religion

There was no significant association between religion and help-seeking behavior, similar to the findings of a study by Valipay et al [5].

Distance from Civil Hospital Valsad

A statistically significant association was found between help-seeking behavior and distance from Civil Hospital Valsad. As the distance from civil hospitals increased, there was a rise in the number of individuals who were seeking treatment late. The majority of the early help seekers were individuals living closer to the hospital. This highlights the need for increasing the availability of psychiatric help nearer to communities to increase its utilization.

Locality

Our study found no association between the locality of participants and their help-seeking behavior. This is consistent with most of the previous studies done previously [23].

Family type

There was no statistically significant association between help-seeking behavior and family type. This is not consistent with the findings of a study by Dew et al. [24], but we did find that, among participants living in nuclear families, the majority (73.68%) were early help seekers. A possible reason for this outcome might be that an individual living in the nuclear family can exercise more independence in seeking help when the need to do so is perceived. Research indicates another reason could be that in joint families, help is only sought when other family members fail to address the problem [5,25].

Socio-economic status

In our study, we found no significant relationship between help-seeking behavior and the socio-economic status of participants. Irrespective of the socio-economic class, the majority of the participants from each class were early help seekers while a study by Gagne et al. suggests that there was a positive association between help-seeking and individuals with low income [26]. There are other studies, for example, one by Gabilondo et al. that found a negative association between individuals with low income and their help-seeking behaviors [25].

Association of severity of illness with help-seeking behavior

In our study, we measured the severity of illness by using the PHQ-9 scale i.e., Patient Health Questionnaire-9. This questionnaire consists of nine questions that are consistent with DSM-IV criteria and each question has a score from 0-3 depending upon the frequency of that symptoms happened within the last two weeks, ‘0’ being ‘not at all,’ to ‘3’ being ‘almost every day’ [15]. We found no significant relationship between the severity and help-seeking behavior of the participants. The course of outcomes in our observation is in line with previous findings done by Lin and Parikh [27] whereas studies done by González et al., suggest that there is a significant association between increasing severity and help-seeking behavior of individuals [18]. But it is noteworthy that at the time of assessing the severity of MDD, many patients were old cases and were already on treatment. This might have affected the outcome.

Reasons for delay in help-seeking

We found a significant relationship between help-seeking behavior and the reasons for help-seeking given by the participants. It is worth noting that the majority of the participants who were late help seekers had a lack of knowledge or awareness about their condition. They were unaware that they were in a state of depression. Another reason for late help-seeking given by participants was the lack of perceived need to go and see a psychiatric doctor. There were individuals with reasons stemming from social stigma like ‘What will other people think?’, or when treatment of other diseases took priority. However, the unavailability of nearby doctors, the unavailability of medicine, or the low quality or lack /limited health care available in the locality impacted both early and late help-seeking behaviors equally. Faith in ayurveda or faith healers showed a positive association with early help-seeking. Another factor that had a major role in determining help-seeking attitudes was that the participants thought that they could cure themselves over time and this is just a phase through which they are going through. Financial reasons also played a major role in help-seeking behavior, but we found a negative association between individuals with higher income and early help-seeking while there was a positive relation between individuals with low income and early help-seeking.

Stigma

We used DSS (depression stigma scale), to measure the stigma of the participants. DSS has two subscales, the personal stigma sub-scale, and the perceived stigma sub-scale. Our study found a statistically significant correlation between personal stigma score and help-seeking behavior, with the mean score negatively associated with early help-seeking. These findings are in line with previous studies done by Boerema et al, who found a negative relationship between higher personal stigma scores and help-seeking [17,19]. This is contrary to the study done by Valipay et al, who found no association between personal stigma and help-seeking but found a significant association between higher mean scores of perceived stigma and help-seeking [5].

Strengths and limitations of the study

The study's strengths lie in its observational cross-sectional design, which provided a cost-effective and efficient means to examine factors influencing health-seeking behavior in individuals with MDD. The representative sample of 102 participants, with a balanced gender distribution and diverse age groups, enhanced the generalizability of the findings. Moreover, the use of validated measurement tools, such as the Patient Health Questionnaire - 9 (PHQ-9) and stigma scores, ensured standardized and reliable assessment of depression symptoms and stigma.

The implications of the study highlight the need for targeted interventions and gender-specific approaches to address barriers to health-seeking behavior among MDD patients. Strengthening primary healthcare centers by providing enhanced training and resources, along with better integration of mental health services, can facilitate early detection and management of MDD. Additionally, awareness campaigns aimed at reducing stigma and increasing knowledge about MDD symptoms and available treatments are crucial to encourage timely help-seeking behavior. Improving accessibility to mental health facilities, particularly in rural areas, can overcome the barrier of distance. Lastly, adopting a multidimensional approach that considers factors like income, marital status, and beliefs can lead to more effective strategies for timely diagnosis and management of MDD. By implementing these implications, it is possible to reduce delays in diagnosis, initiate appropriate treatment, and improve the prognosis for individuals with MDD.

The limitation of this study was that it was a hospital-based study, hence patients who are not seeking health care and who have discontinued the treatment could not be included. This study can be replicated in a community setting to get a more accurate representation of the help-seeking behavior of people suffering from MDD.

## Conclusions

There was a significant association between gender and help-seeking behavior with female participants being early help seekers while more male participants were late help seekers. There was a significant association between marital status and help-seeking behavior of the study participants with marriage playing a positive role in early help-seeking. An increase in the distance between the Civil Hospital Valsad and the participants' homes was negatively associated with help-seeking behavior. We discovered there was a statistically significant association between early and late help-seeking behavior and mean personal DSS score, with the scores being higher in late help-seekers indicating higher personal stigma.

There was no statistically significant relationship found between the participant’s age group, educational level, location, family type, socioeconomic class, disease severity, perceived stigma, and help-seeking behavior. The major reasons found to be responsible for delayed treatment seeking were lack of awareness, wrong beliefs about getting cured on their own, social stigma, and financial reasons.

## References

[1] (2023). WHO. Depression. https://www.who.int/news-room/fact-sheets/detail/depression.

[2] Gururaj G, Varghese M (2016). National Mental Health Survey of India, 2015-16: Prevalence, Patterns and Outcomes. http://indianmhs.nimhans.ac.in/Docs/Report2.pdf.

[3] Cui R (2015). Editorial: a systematic review of depression. Curr Neuropharmacol.

[4] Ferrari AJ, Somerville AJ, Baxter AJ, Norman R, Patten SB, Vos T, Whiteford HA (2013). Global variation in the prevalence and incidence of major depressive disorder: a systematic review of the epidemiological literature. Psychol Med.

[5] Valipay SK, Parikh MN, Desai M, Nathametha BT (2019). A study of factors affecting help-seeking behavior in major depressive disorder. Annals of Indian Psychiatry.

[6] Doblyte S, Jiménez-Mejías E (2017). Understanding help-seeking behavior in depression: a qualitative synthesis of patients’ experiences. Qual Health Res.

[7] Roberts T, Shidhaye R, Patel V, Rathod SD (2020). Health care use and treatment-seeking for depression symptoms in rural India: an exploratory cross-sectional analysis. BMC Health Serv Res.

[8] Roberts T, Shrivastava R, Koschorke M, Patel V, Shidhaye R, Rathod SD (2020). "Is there a medicine for these tensions?" Barriers to treatment-seeking for depressive symptoms in rural India: A qualitative study. Soc Sci Med.

[9] Devarapalli SV, Kallakuri S, Salam A, Maulik PK (2020). Mental health research on scheduled tribes in India. Indian J Psychiatry.

[10] Andersen RM (2008). National health surveys and the behavioral model of health services use. Med Care.

[11] GUJARAT SERIES-25 PART XII-B DISTRICT CENSUS HANDBOOK VALSAD VILLAGE AND TOWN WISE PRIMARY CENSUS ABSTRACT (PCA (2023). Census of India 2011 - Gujarat - Series 25 - Part XII B - District Census Handbook, Valsad. India.

[12] Gururaj G, Girish N, Isaac MK (2023). NCMH Background Papers-Burden of Disease in India. Mental, neurological and substance abuse disorders: Strategies towards a systems approach. http://cdrwww.who.int/entity/macrohealth/action/NCMH_Burdenofdisease.

[13] (2023). Census 2011. Valsad District - Population 2011-2023. https://www.census2011.co.in/census/district/205-valsad.html.

[14] Costantini L, Pasquarella C, Odone A (2021). Screening for depression in primary care with Patient Health Questionnaire-9 (PHQ-9): A systematic review. J Affect Disord.

[15] (2023). Stanford Medicine. Patient Health Questionnaire-9 (PHQ-9). https://med.stanford.edu/fastlab/research/imapp/msrs/_jcr_content/main/accordion/accordion_content3/download_256324296/file.res/PHQ9%20id%20date%2008.03.pdf.

[16] Griffiths KM, Christensen H, Jorm AF (2008). Predictors of depression stigma. BMC Psychiatry.

[17] Boerema AM, Zoonen Kv, Cuijpers P, Holtmaat CJ, Mokkink LB, Griffiths KM, Kleiboer AM (2016). Psychometric properties of the Dutch Depression Stigma Scale (DSS) and associations with personal and perceived stigma in a depressed and community sample. PLoS One.

[18] González HM, Vega WA, Williams DR, Tarraf W, West BT, Neighbors HW (2010). Depression care in the United States: too little for too few. Arch Gen Psychiatry.

[19] Boerema AM, Kleiboer A, Beekman AT, van Zoonen K, Dijkshoorn H, Cuijpers P (2016). Determinants of help-seeking behavior in depression: a cross-sectional study. BMC Psychiatry.

[20] Sussman LK, Robins LN, Earls F (1987). Treatment-seeking for depression by black and white Americans. Soc Sci Med.

[21] Coryell W, Endicott J, Winokur G (1995). Characteristics and significance of untreated major depressive disorder. Am J Psychiatry.

[22] Magaard JL, Seeralan T, Schulz H, Brütt AL (2017). Factors associated with help-seeking behaviour among individuals with major depression: A systematic review. PLoS One.

[23] Mackenzie CS, Pagura J, Sareen J (2010). Correlates of perceived need for and use of mental health services by older adults in the collaborative psychiatric epidemiology surveys. Am J Geriatr Psychiatry.

[24] Dew MA, Bromet EJ, Schulberg HC, Parkinson DK, Curtis EC (1991). Factors affecting service utilization for depression in a white collar population. Soc Psychiatry Psychiatr Epidemiol.

[25] Gabilondo A, Rojas-Farreras S, Rodríguez A (2011). Use of primary and specialized mental health care for a major depressive episode in Spain by ESEMeD respondents. Psychiatr Serv.

[26] Gagné S, Vasiliadis HM, Préville M (2014). Gender differences in general and specialty outpatient mental health service use for depression. BMC Psychiatry.

[27] Lin E, Parikh SV (1999). Sociodemographic, clinical, and attitudinal characteristics of the untreated depressed in Ontario. J Affect Disord.

